# Optimising co-design processes in telemedicine innovation–developing a telemedical solution for emergency medical services

**DOI:** 10.1371/journal.pone.0309955

**Published:** 2024-10-18

**Authors:** Elisabeth Klager, Josef Michael Lintschinger, Anna Teufel, Eva Schaden, Valerie Manschein, Lena Reischmann-Senoner, Stefan Ulbing, Harald Willschke, Christoph Frimmel, Reinhold Renner, Christiane Grill, Christina Hafner

**Affiliations:** 1 Ludwig Boltzmann Institute Digital Health and Patient Safety, Vienna, Austria; 2 Department of Anaesthesia, Intensive Care Medicine and Pain Medicine, Medical University of Vienna, Vienna, Austria; 3 Austrian Red Cross, State Association of Burgenland, Austria; 4 Open Innovation in Science Center, Ludwig Boltzmann Gesellschaft, Vienna, Austria; Najran University College of Computer Science and Information Systems, SAUDI ARABIA

## Abstract

**Background:**

Stakeholder engagement plays a vital role in driving advancements in product development. This imperative now extends to the healthcare domain, driven by the scarcity of healthcare professionals and the pressing demand for effective solutions. Through the application of design thinking and co-design methodologies, this study endeavours to promote comprehensive stakeholder involvement, creating streamlined processes and adaptable templates geared towards fostering innovative solutions for tele-emergency medicine.

**Methods:**

In this study design thinking and co-design methods are developed, adapted, and tested, to create effective tools and demonstrate their application. This is part of a process involving stakeholders and lead users to develop a telemedicine solution for emergency medical services. This research is descriptive in kind, offering a transparent and holistic portrayal of the co-design process. The rural region of Burgenland in Austria was chosen for this study, with the challenges of its weak infrastructure offering valuable insights. The tools were tested in co-design workshops, with the participants continuously observed by the research team.

**Results:**

Seventeen healthcare professionals, emergency medical technicians and academics participated in a co-design process to develop a telemedicine solution for emergency medical services. The results section presents practical co-creative healthcare innovation tools and templates that have been shown to facilitate the co-design process.

**Conclusions:**

The study developed and applied co-design elements for the creation of a prototype concept for telemedicine in emergency medicine and offers valuable insights for similar projects involving diverse stakeholders. It shows that structured co-design activities help all stakeholders to jointly create solutions that meet the overall needs.

## Introduction

Involving stakeholders in development processes is routine practice in many industrial fields. It has been shown that user co-designed and co-developed products and solutions are more compelling, feasible, and viable [1–3]. In recent years, this trend has also reached the healthcare sector, with patients and healthcare professionals (HCP) now seen as indispensable to development processes and valued as active partners and sources of innovation [1]. Although those approaches are time-consuming, it is a worthwhile methodology known to have a positive effect on the rigour, relevance, and reach of a project, as well as the future implementation process [4, 5]. Innovation science distinguishes between different levels of involvement of users in the development process. In user-centered design, the perspectives, needs, and expectations of users are carefully considered, yet their direct involvement in the development process is not mandatory [6]. Contrastingly, employing co-design processes within a participatory framework necessitates genuine user involvement, positioning them as active co-designers, thereby fostering a deeper level of engagement [3]. More recent studies proved that stronger involvement of users and stakeholders in the co-design process leads to higher satisfaction while using the resulting application [7, 8]. Moreover, within the present model-driven software engineering paradigm in the industry, the integration of co-design and collaborative efforts with users stands as indispensable for achieving successful outcomes [9].

Stakeholder and user engagement is particularly important in complex environments in which new solutions are urgently needed: Healthcare is a prime example as it involves a diverse group of stakeholders including policy makers, social insurance providers, HCP, general practitioners, hospitals, etc. Worldwide, the increasing shortage of HCP is placing an enormous burden on healthcare systems, especially prehospital emergency medicine. In Germany and Austria, especially in rural areas, prehospital emergency medicine is facing a steady rise in missions paired with a scarcity of emergency physicians (EPs) [10, 11]. Around 10 years ago, an innovative pilot was started in Aachen in Germany, implementing telemedicine for prehospital emergency medicine. It showed that telemedicine could be a viable solution for HCP facing a scarcity of prehospital emergency medical personnel, by offering access to an additional source of expertise and a second opinion for less experienced EMTs on scene [12]. Although there have been very strong developments in this area in recent years, a lot of projects involved in transferring telemedical solutions into routine care fail due to a lack of consideration given to the stakeholders and change management aspects [13, 14]. Furthermore, the potential for prehospital care in particular appears to be untapped [15]. Best practice solutions in Germany recommend involving all relevant stakeholders right from the start to successfully develop a telemedicine solution and achieve high levels of stakeholder acceptance [12]. This is consistent with the theory of open innovation in science and ideas of design thinking, co-design, participatory design and user-centred design, all systematic, iterative, and exploratory processes of innovative problem solving [16, 17]. One key reason why design thinking is seen as particularly valuable is its focus on empathy amongst all those involved [18] and its approach of developing a solution from the standpoint of the user [19]. The first of five phases of design thinking involves carefully examining the needs, perceptions, expectations, and challenges, before clearly defining the problem in the second phase. Ideation methods are applied in the third phase, followed by a fourth phase of prototyping, and the final fifth phase, testing. These phases are not linear but evolve as iterative elements [1, 20].

In the current scientific literature, there are several studies on sub-elements of co-creative processes in healthcare. However, few studies clearly describe and accompany the entire process from needs analysis to prototype, which is also described as a particular concern of researchers [21]. This is especially true for the field of emergency medical services (EMS). This research paper is thus part of a long-term project. In a first experimental project phase, we used a mixed-methods approach to delve into stakeholder perceptions, needs, and challenges in the pilot area of Burgenland, a federal state of Austria. Data from this previous study are available on QDR [22].

Using these previous findings, the aim and novel aspect of this present study is to employ design thinking and co-design in a thoughtful combination of evidence-based approaches in order to actively engage stakeholders and users in the complete process of developing a telemedicine solution for EMS in a rural area. The ultimate project goal was to provide guidelines for other research projects and research teams, detailing the process and providing readily implementable templates that have been shown to facilitate co-design processes.

## Materials and methods

### Setting: Emergency medical service in rural Austria

This study was conceptualised as a descriptive study using the methodology of co-design, participatory design, and elements of implementation science in the tradition of open innovation in science. Some of the methods and results are presented jointly for the purposes of readability.

The region of Burgenland in Austria was selected as the ideal setting for this study due to its unique characteristics. With a notably weak infrastructure, considerable distances between hospitals, and lower deployment figures, Burgenland offers valuable insights for rural regions.

**Fig 1:**
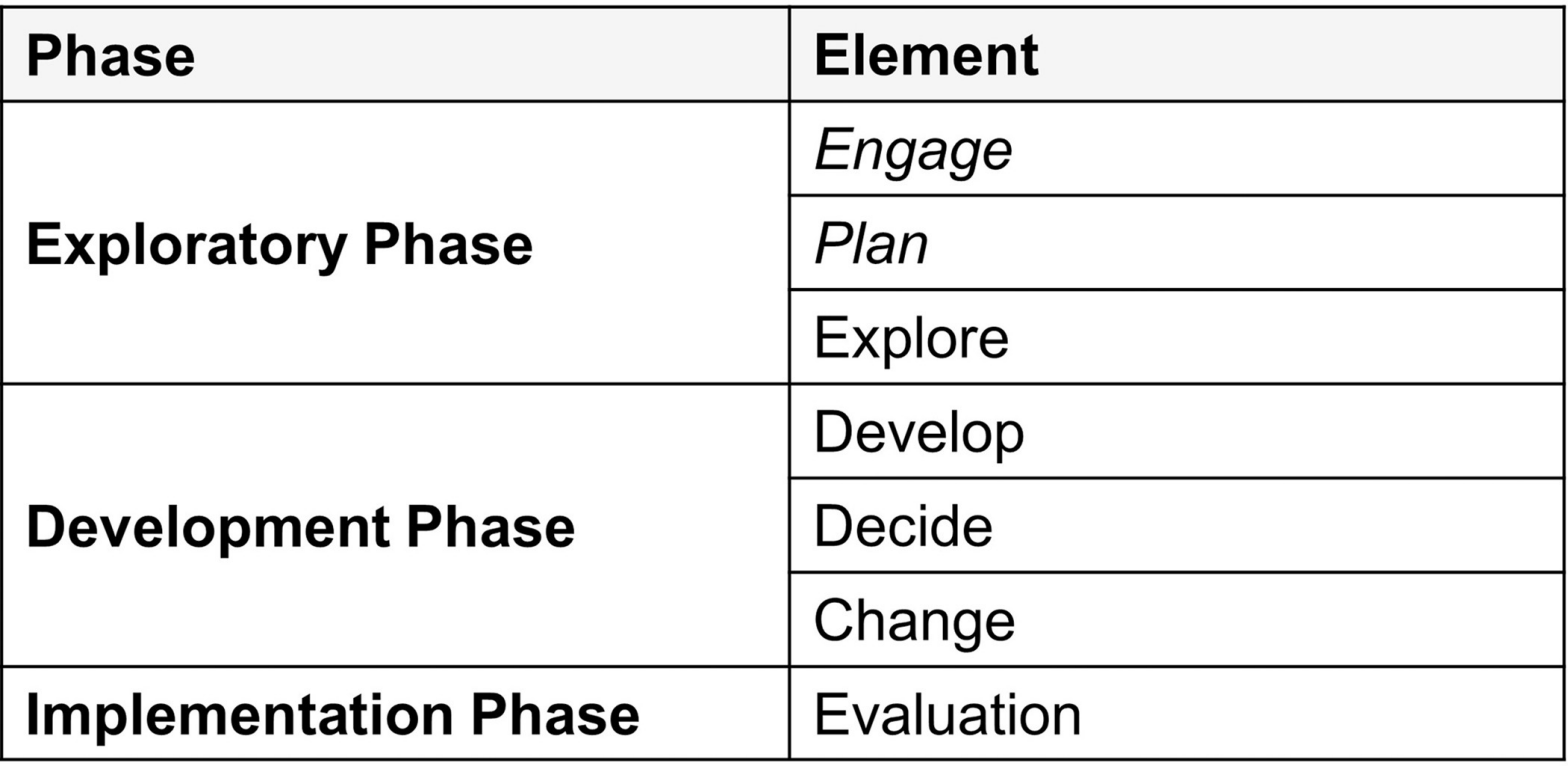
Framework of co-design by Talevski et al.

This study builds on an earlier study within the same project in which focus groups and quantitative questionnaires were used to survey stakeholder expectations and concerns in the region of Burgenland [22]. Following the existing principles and frameworks of co-design [20], we decided to build on the model of Talevski et al. [23] Our first study covered the phases of “Engage & Plan”, while the current study consists of “Explore & Develop”. The third and next project phase (not part of this paper) will encompass “Decide & Change” [23] (Fig 1).

### Our approach to co-design

Our study aims to define the optimal processes, methods and tools for jointly developing a telemedicine solution for the EMS in the pilot region [24] using participatory design. Taking Talevski et. al. as a basis, we iteratively selected and created methods and tools for the co-design workshop.

### Participants and planned contribution

**Fig 2:**
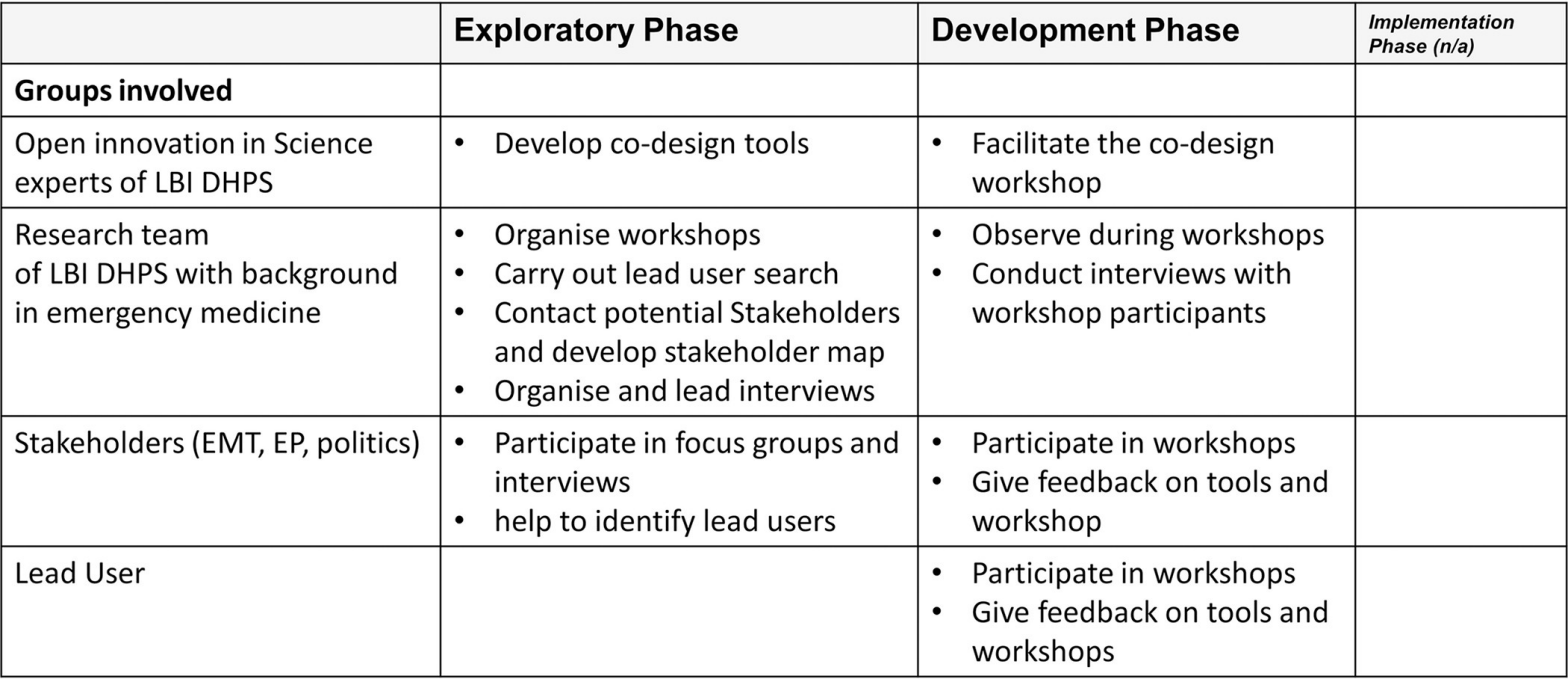
Level of contribution by the different groups involved.

In the current study, we focused on the involvement of relevant stakeholders as well as lead users (individuals or organisations exhibiting early and innovative solution adoption and often serving as a source of valuable insights and feedback for further development) in order to learn from the best [25]. To create a stakeholder map, relevant players needed to be identified via internet research, personal contacts, and presence at relevant conferences and events, and then, in a next step, presented in the form of a clear stakeholder map and a respective list. In the rural region of Burgenland, relevant stakeholders (i.e., HCP, volunteer EMTs, legal representatives and academics) were involved in co-creating a telemedicine solution for emergency medicine right from the start. Applying the theory of lead users [25], the next step was a lead user search, using literature search and direct contacts, enabling us to identify experienced and knowledgeable experts in the German-speaking field of tele-emergency medicine (due to the similarity of the healthcare systems), to learn from the best and build on their previous experience. In order to provide ample opportunity for a participatory approach, the specific phases during which stakeholders could contribute and the extent of their involvement was carefully planned (Fig 2).

Seventeen participants from Austria travelled to Aachen in Germany where a best practice example of tele-EMS has been in operation for several years. This gave them the opportunity to experience firsthand how a tele-EMS solution can work and to take part in a 2-day interdisciplinary co-design workshop. Four of the participants were from the research team of the Ludwig Boltzmann Institute Digital Health and Patient Safety (LBI DHPS) and had a background in (emergency) medicine; one was a facilitator from LBI DHPS; nine were from the Austrian Red Cross in Burgenland; two from the emergency communications centre in Burgenland; and one with a political background who is active in local health politics.

Prior to the excursion to Aachen, several coordination and preparatory meetings were conducted by the project team so that the development process could be built on prior knowledge and a good common basis. The workshop in Aachen consisted of two thematic focuses: learning from experience and creating something new and tailor-made for the selected area. The first day of the workshop focused on learning from the group members’ past experiences, and identifying the contribution that each workshop participant could make to the project. The second workshop day applied the carefully prepared co-creation tools for the whole group, moderated by a facilitator from the LBI DHPS.

### Development of tools and methods for the co-design workshop

**Fig 3:**
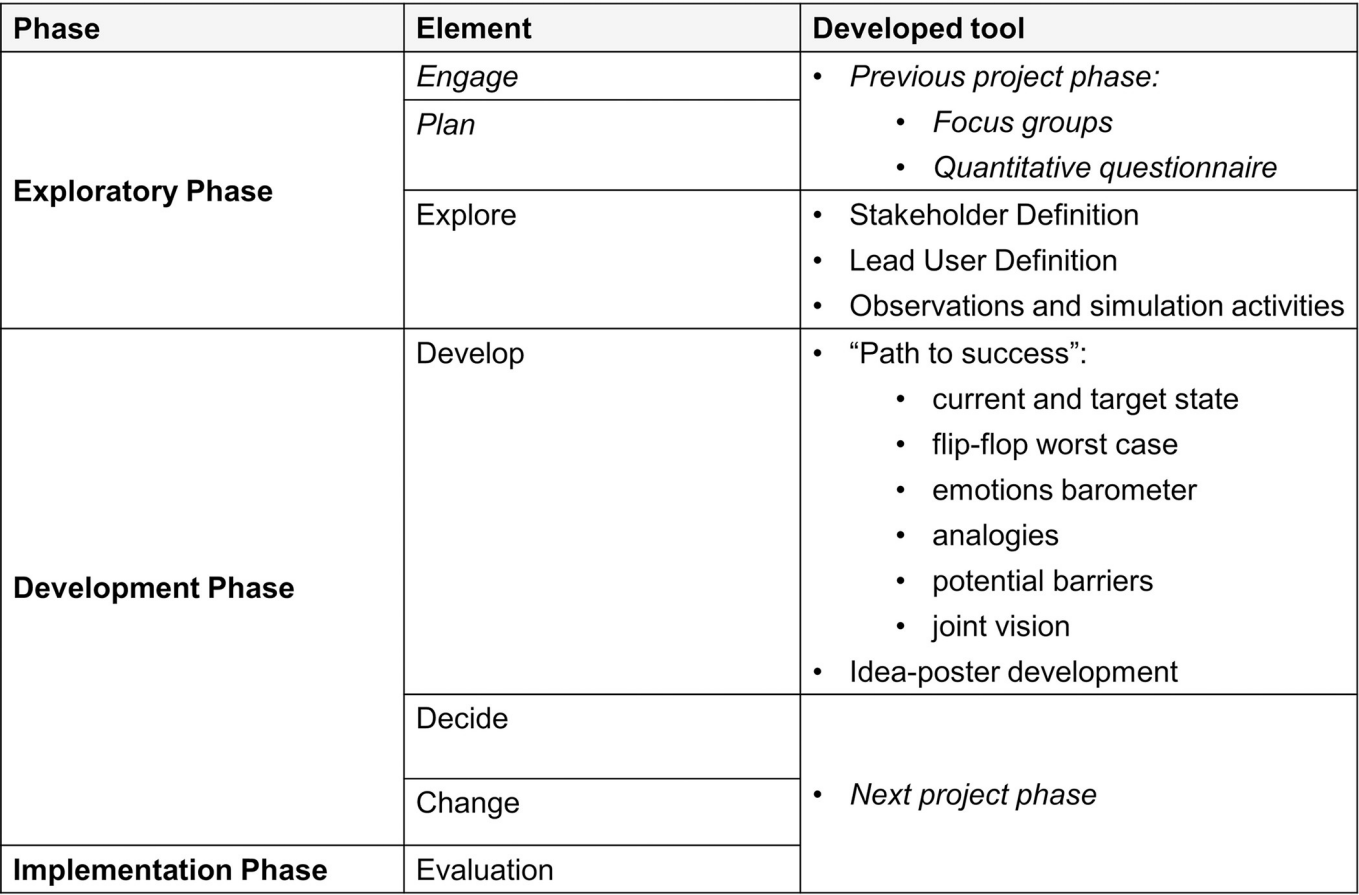
Set of tools for the co-design process.

To select tools and methods, we started with a review of the current literature on design thinking, participatory design, and co-design in healthcare with the intention of identifying and prioritising those tools and templates most useful for the intended development process. This was executed by the research team in collaboration with academics and project managers at the Open Innovation in Science Center at the Ludwig Boltzmann Gesellschaft. During this process we familiarised ourselves with design-thinking toolkits for healthcare innovation, e.g., by Johnsen et. al. [19] and identified frameworks by Eppler [26]. We then adapted the selected frameworks and created individualised templates (Fig 3).

**Fig 4:**
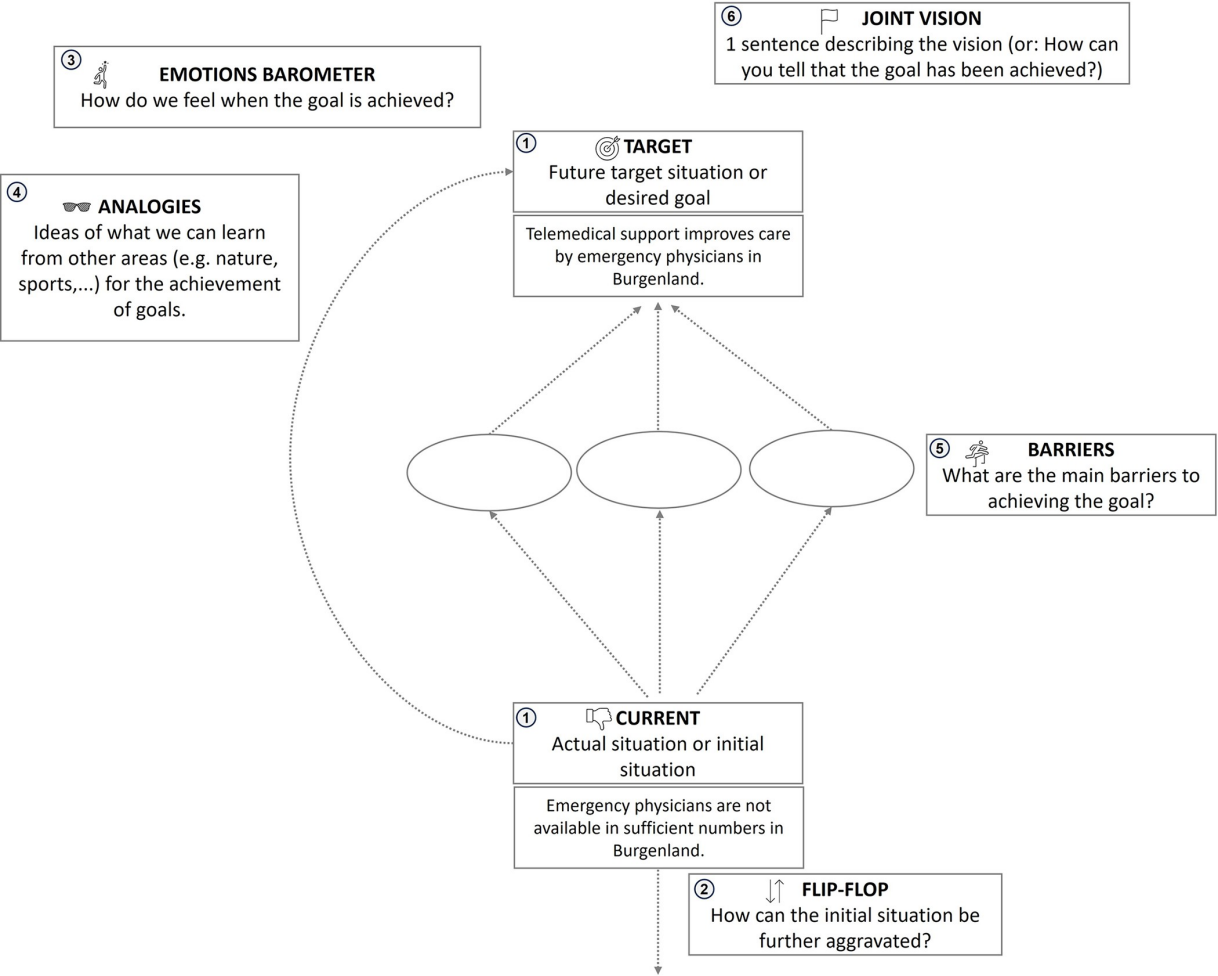
Set of tools used in the workshop, co-developed with the Open Innovation in Science Center at the Ludwig Boltzmann Gesellschaft, based on the ideas of Eppler 2013.

The main template was prepared for printing on a large poster and consists of several tools allowing the ideation process to be viewed from various angles (Fig 4). The tools were used in the order shown below. In the workshop, the moderator first explained the tools before asking the participants to discuss the associated questions and to document their thoughts and contributions on post-it notes.

### The prepared tools are as follows

“Current vs target state”: In this tool, the participants discuss the question “What is the current situation?” and define the challenges, needs and positive aspects of the status quo in their own words. They compare this defined status with the target situation that they want to achieve with this project.

“Flip-flop worst case scenario”: This tool prompts participants to envision and articulate a scenario that exacerbates the current situation. Participants are tasked with describing the situation in its most dire manifestation and recording these thoughts on post-it notes.

“Emotions barometer”: In this task, the workshop participants are asked to imagine that they have achieved their goal and to describe their feelings in this state.

“Finding analogies”: This tool encourages participants to think about analogies to the strengths needed here. They are asked to consider what they might need to master the current issue or challenge and how it might be possible to learn from other areas.

“Potential barriers”: As the name suggests, this task is about naming all possible barriers that could stand in the way of the project and then specifying the three most important ones in a group discussion.

“Joint vision”: The participants are asked to formulate how they might recognise that their goal has been achieved in a single sentence. This sentence was then adopted as their shared vision for the project.

**Fig 5:**
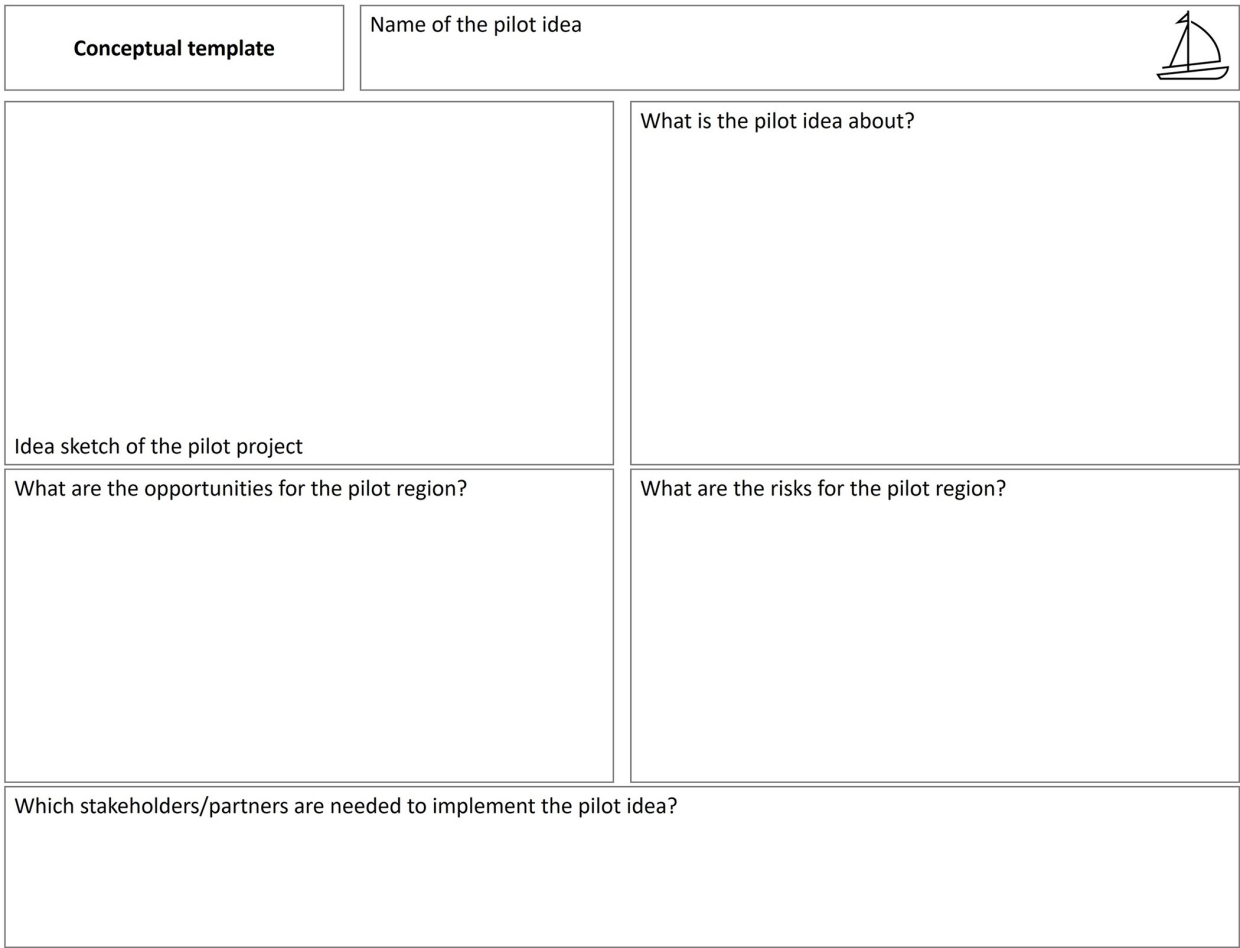
Conceptual template used in the workshop, co-developed with the Open Innovation in Science Center at the Ludwig Boltzmann Gesellschaft based on the ideas of Eppler 2013.

The workshop participants worked with the set of tools shown above to derive an initial goal. The group then divided into two small heterogeneous groups, each of which sketched out their concrete idea for a pilot using a prepared idea poster template (Fig 5).

### Process evaluation

The entire process of the 2-day workshop was accompanied scientifically to observe the effects of the developed and applied tools. Our defined endpoints were the perceived usefulness of the tools and their supposed impact on the process. Three researchers from the LBI DHPS used self-designed observation protocols to make field notes and interview participants, and then noted their insights as detailed observation protocols. This ensured all process steps were recorded and, above all, that the reactions of the participants were described on a continuous basis.

Our results therefore present the developed tools and the rationale behind their use, and also show the results we achieved when applied in the tele-emergency medicine setting.

We received an exemption from the approval requirement by the Chairman of the Ethics Committee for the Vienna Hospitals in Vinzenz Holding for this study. This exemption is in accordance with Austrian law and the principles of the Declaration of Helsinki as well as the General Data Protection Regulation of the European Union (GDPR).

## Results

The results section describes the effect and rationale of the tools used to clarify their practical application while also describing their observed effect. We also describe their planned and actual role in facilitating the process. The processes and templates are the respective results of our preparatory work based on the co-design model of Talevski et al. and Eppler et al. [23, 26]. They can be adapted to specific needs and used by those wishing to involve relevant stakeholders and users in design and development processes.

### Stakeholder definition and lead user identification

In our stakeholder map, we identified 12 stakeholders in Burgenland (Austria) who work in or with EMS and are ideal participants for the further co-design process: They appeared open to new solutions; act in a reflective and realistic manner; and can play a role in shaping the implementation of new solutions in the region. Our lead user search quickly led us to tele-EMS Aachen in Germany, a highly experienced team that introduced tele-emergency medicine as early as 2014. The tele-EMS Aachen team submitted a list of experts with details of their respective backgrounds and know-how to our project team. The five identified lead users of the tele-EMS Aachen team joined the stakeholders from Burgenland on the 2-day excursion to the tele-EMS headquarter in Aachen.

### Co-design workshop

The planned co-design workshop was scheduled for two days, with a specific focus on getting to know each other and building an atmosphere of trust. Preparatory calls and meetings between all planned workshop participants facilitated a smooth communication process. The visit to the tele-EMS Aachen headquarters was particularly valuable, offering the chance to observe live operations and take part in several simulations. This was key to creating a shared understanding of the opportunities and challenges of telemedicine in emergency services, with a particular focus on the necessary skills and interpersonal relationships. The second day was led by a facilitator from LBI DHPS who moderated the workshop and applied the prepared co-creation tools.

The next section describes the results for each tool on two levels: firstly, the reasons for using the tool and its demonstrated impact on the process, and secondly, the substantive results for the EMS setting in Burgenland.

### Tool “Current vs target state”

Working with this tool took 20 minutes. We observed that by using this tool, all workshop participants were quickly able to achieve a joint understanding of the target state and thus establish an excellent basis for starting their work.

The discussions about current and target state showed very clearly that there is currently insufficient provision of EPs in the pilot region. It was also frequently highlighted that the quality of emergency medicine training is a key factor for such a system: only well-trained EMTs can put the tele-expertise provided by EPs into practice during patient care. The target state was jointly described as a system in which telemedical support would improve the supply of emergency medical services in the region of Burgenland.

### Tool “Flip-flop worst case scenario”

The participants worked with the idea of worst-case scenarios for 15 minutes. It became obvious that this tool invited all participants to openly bring their concerns to the table as it actively encouraged them to imagine the worst case. It was also observed that this tool functioned as an icebreaker.

During the process of considering a worst-case scenario for the situation of EMS in Burgenland and exploring factors which might exacerbate the situation, various concerns were raised. These included even more acute shortages, inadequate training, and extended service times resulting in less patient safety.

### Tool “Emotions barometer”

To form an arc encompassing everything from the worst-case scenario through to a positive outcome, this tool was used for 10 minutes to add an emotional perspective and increase identification with the topic. As with the previous tool, the request to formulate emotions enabled everyone to participate and articulate their own thoughts. This noticeably strengthened the personal connection to the topic and the group.

The entire group used this tool to discuss the positive emotions that would be felt in the event of a successful solution. These covered everything from a feeling of security, to motivation, euphoria, pride, and encouragement, and the satisfaction of having solved a problem.

### Tool “Finding analogies”

The “Finding analogies” tool was conducted in 15 minutes and was used to encourage participants to consider the “big picture”. We observed that using this tool helped participants think more widely, think “out of the box”, leaving well-travelled thought pathways to create new perspectives.

Participants in the workshops developed ideas including symbolic images from sport to represent a strong team spirit, and discussions about the need for training and experience to achieve success–a process that is “not a sprint, but a marathon”.

### Tool “Potential barriers”

This tool was used for 20 minutes to create awareness of the potential roadblocks that need to be addressed at an early stage, and to focus on the most important ones. We have found that openly naming barriers contributes to greater transparency and clarity, and has brought greater openness and understanding overall for the individual participants.

In the workshop the participants identified three main barriers, namely insufficient funding; training complexities; and skills shortages. Other obstacles noted include limited acceptance, potential apprehensions, insufficient political commitment, and legal constraints.

### Tool “Joint vision”

Using the “joint vision” tool, the participants were given 20 minutes to define a common goal. Thanks to the rigorous and clear process and the previous steps that had put everyone on the same page, this vision was committed to paper quickly and precisely. One participant argued that “involving stakeholders could be instrumental to ensuring that they act as multipliers in carrying the jointly developed ideas into the organisations, thereby ensuring greater acceptance”.

In the workshop the participants agreed on the following vision: An evidence-based framework should be jointly created, a 1 to 2-year project set up, and the number of requests via the tele-emergency physician, the physician-free intervals, the changes concerning target hospitals, and the change in diagnosis and treatment on-site all recorded and clearly analysed.

### Tool “Idea poster”

Working in two heterogeneous subgroups for 90 minutes, two different ideas were developed and sketched out by the workshop participants as they followed the “idea poster” template. The clear template and strict timing supported a constructive ideation process and precise description of the emerging ideas.

During this workshop process two ideas emerged: “Tele-Doc-Service Burgenland” and “Koarl”. Despite subtle differences, the two ideas can be easily combined for the planned pilot project. They are based on the idea that a simple telemedical solution involving headset, smartphone, and data transmission capability, paired with a standardised documentation/transfer protocol, will be used on rescue missions to secure access to expertise and a second opinion. The focus of the pilot project will be to prove feasibility, collect data, expand current medical availability, and conduct a financial evaluation. Politicians, social insurance carriers and various rescue organisations were specified as necessary stakeholders for the next steps.

One participant expressed their astonishment at the outcome, stating, “I would not have imagined that we could make so much progress in just two days by having everyone sitting at the same table.” Another participant noted with surprise that “the well organised and well-prepared co-design tool mix and the pleasant atmosphere significantly reduced scepticism among the participants.” Interestingly for the participants, in terms of content, during the process they clearly abandoned the idea that image transmission is essential. Without the simulations and conversations with the lead users, they would have probably made this central to the concept, whereas they now regard this aspect as unimportant.

It was crucial to involve stakeholders and end-users–in this case EPs and EMTs–in the process because of their deep understanding and knowledge of the relevant environment. Involving them at an early stage led to a high level of engagement, despite some initial scepticism.

## Discussion

Within the scope of this article, we provide a transparent description of the co-design process and share ready-to-use-templates. Furthermore, we give insights into the content developed in the workshop for a telemedicine solution in emergency medical services in Austria. This paper outlines a theory-based process of how to involve stakeholders in the co-design of a telemedicine solution and offers templates that can be adapted and used for similar projects. While much more commonly used in other industries, the methodology of co-design and design thinking has only recently found its way into the healthcare system. In a recent literature review, Pühvel et al. used the search words “Design thinking”, “Intervention”, and “Health” to identify 40 articles published between 2020 and 2022 [27] which describe how different studies and fields in healthcare have used methods of design thinking and related tools for respective project phases. Our project was able to build on the methods covered in the Pühvel review, i.e., literature reviews, focus group discussion, brainstorming processes, collecting ideas into cohesive concepts, detailing them, and explaining the reason for our choices. The novelty of our project was to plan and thoroughly execute a holistic process starting from needs definition, whereas to date studies have often focused on single elements and examined individual process steps. In line with previous studies, we discovered that applying a co-design approach can be time consuming. However, we succeeded in developing a holistic idea that would not have been possible without the involvement of a wide range of stakeholders contributing their experiences and opinions [28].

In the co-design workshop in Aachen, participants realised that by using methods of co-design it was possible to go beyond the standard methods of problem solving to find better, more suitable, and satisfying solutions, as suggested by Roberts et al. [1]. This was a particularly satisfying finding. One of our lead users explained that a key learning from their experience with telemedicine in Aachen is that involving all stakeholders from the beginning would have simplified the process and reduced barriers and challenges. As previous research has shown, it is therefore essential to start involving stakeholders at a very early stage so that their experiences and needs can be effectively incorporated from the outset [29–31]. If digital solutions such as telemedicine do not meet the expectations of healthcare professionals, it is likely that the implemented tool will not be used as anticipated [32]. As shown in the literature, we also confirmed that a workshop involving a heterogeneous mix of participants in a pleasant and enjoyable atmosphere is of immeasurable importance to the outcome, as is ensuring that the participation is meaningful for all those who engage [33]. As a prerequisite on an individual level, participants in co-design processes need to cultivate the virtues of cooperation, curiosity, creativity, empowerment and reflexivity, as previously also argued by Steen et al. [34]. Being aware of these essential pre-conditions, our project team set great store by clear, iterative, and transparent communication and the development of interesting and entertaining methods and tools. This was highly appreciated by the participants according to the reported experiences throughout the project.

In the descriptions of their methods, several co-design reviews fail to explain the co-design activities in sufficient detail, with the result that it is not possible to determine their actual content or to replicate them [21]. Slattery et al. express a desire both for the use of clearer and more consistent terminology (although this has improved in recent years, e.g., in publications by Talevski, Boyd, etc.) and for more precise reporting of the activities involved [21]. Their second request has been carefully considered in this study, namely that the templates for the co-design elements are presented and the reasons for their use explained in detail [26].

It is clear that on a global level the shortage of healthcare staff is becoming a key challenge that healthcare must master [35]. Given current pressures on resources and the shortage of healthcare professionals, participation and co-design could be useful tools for making careers in healthcare more attractive, offering a means by which their skills, knowledge, and expertise can be applied to shape the future of the healthcare profession [36]. As our study has shown, the respective stakeholders had a strong desire to be involved, to be part of the development process to drive change, and to be part of the solution. We were also able to show that the stakeholders felt that they achieved better results as a diverse group, which is supported by the scientific literature on user-centredness and co-design (1).

### Implications for co-design research

The novelty value of our study lies in providing application-oriented guidance in a holistic process that is easy to implement. While our results are based on innovation science theories, they are also extremely practical as they offer tools and frameworks for projects in similar situations in which stakeholder involvement and joint development is key. Our study deliberately describes the co-activity templates in detail and explains the rationale behind their use so that they can also be applied by others in related fields.

### Outlook and next project steps

After completion of the “develop” phase, a concrete and detailed concept will be written up and developed further in close cooperation with all stakeholders and, in a next step, sent to decision makers in the pilot region for planning the implementation and scientific monitoring.

### Strengths and limitations

This paper is based on evidence from innovation science and has carefully selected and adapted tools. Consequently, it highlights the usefulness of stakeholder involvement. The main limitation is that it has only been tested for the particular use case of co-designing a tele-emergency solution. Furthermore, the methodological approach was rather descriptive including observations and unstructured interviews instead of a functional analysis and correlation with potential outcomes. While the results that emerged directly from the development process will be less transferable to other areas and regions, the templates and methods themselves are applicable in completely different settings. Further studies could focus on addressing this limitation by adapting the same tools to a different set-up in order to evaluate any differences.

## Conclusions

This study brought the co-design elements to life and yielded highly satisfactory results for stakeholders, confirming the value of the participatory approach with stakeholders and lead users. We also provide a clear and holistic process with methods and tools that can serve as a useful guide for similar implementation projects involving a variety of different stakeholders in healthcare. With regard to our project, the next steps will include deploying the pilot as well as a deeper focus on the patient perspective, to gain insights into the patient experience of telemedical support in emergency medicine.
